# A dicoumarol-graphene oxide quantum dot polymer inhibits porcine reproductive and respiratory syndrome virus through the JAK-STAT signaling pathway

**DOI:** 10.3389/fmicb.2024.1417404

**Published:** 2024-06-19

**Authors:** Zhuowei Li, Junjun Wang, Siyu Wang, Wei Zhao, Xiaolin Hou, Jianfang Wang, Hong Dong, Shuanghai Zhou, Yuan Gao, Wei Yao, Huanrong Li, Xuewei Liu

**Affiliations:** ^1^College of Animal Science and Technology, Beijing University of Agriculture, Beijing, China; ^2^Beijing Key Laboratory of Traditional Chinese Veterinary Medicine, Beijing Traditional Chinese Veterinary Engineering Center, Beijing University of Agriculture, Beijing, China; ^3^Liaoning Agricultural Development Service Center Province, Shenyang, China

**Keywords:** PRRSV, dicoumarol, graphene oxide quantum dot polymer, antiviral, JAK/STAT signaling pathway

## Abstract

**Introduction:**

Porcine reproductive and respiratory syndrome virus (PRRSV) causes substantial economic losses in the global swine industry. The current vaccine options offer limited protection against PRRSV transmission, and there are no effective commercial antivirals available. Therefore, there is an urgent need to develop new antiviral strategies that slow global PRRSV transmission.

**Methods:**

In this study, we synthesized a dicoumarol-graphene oxide quantum dot (DIC-GQD) polymer with excellent biocompatibility. This polymer was synthesized via an electrostatic adsorption method using the natural drug DIC and GQDs as raw materials.

**Results:**

Our findings demonstrated that DIC exhibits high anti-PRRSV activity by inhibiting the PRRSV replication stage. The transcriptome sequencing analysis revealed that DIC treatment stimulates genes associated with the Janus kinase/signal transducer and activator of transcription (JAK/STAT) signalling pathway. In porcine alveolar macrophages (PAMs), DIC-GQDs induce TYK2, JAK1, STAT1, and STAT2 phosphorylation, leading to the upregulation of JAK1, STAT1, STAT2, interferon-β (IFN-β) and interferon-stimulated genes (ISGs). Animal challenge experiments further confirmed that DIC-GQDs effectively alleviated clinical symptoms and pathological reactions in the lungs, spleen, and lymph nodes of PRRSV-infected pigs.

**Discussion:**

These findings suggest that DIC-GQDs significantly inhibits PRRSV proliferation by activating the JAK/STAT signalling pathway. Therefore, DIC-GQDs hold promise as an alternative treatment for PRRSV infection.

## 1 Introduction

Graphene oxide quantum dots (GQDs) are a type of nanomaterial that has gained increasing attention in recent years due to their unique properties and potential applications in various fields (Liu et al., 2013). GQDs have a size range of 1–10 nm and exhibit excellent biocompatibility, low toxicity, and high stability. These characteristics make them highly suitable for biomedical applications, including drug delivery and bioimaging (Sun et al., 2013; Zheng et al., 2015; Vahedi et al., 2022). GQDs can be easily functionalized with biomolecules such as proteins, DNA, and antibodies, enabling targeted delivery and imaging of specific cells or tissues (Haider et al., 2023). While there have been multiple reports on the inhibition of PRRSV proliferation by graphene oxide, there is a lack of research on the effect of GQDs on PRRSV (Guo et al., 2019; Liu X. et al., 2022).

Dicoumarol (DIC) is a natural compound that is mainly found in plants of the Rutaceae, Umbelliferae, and Compositae families. It was originally extracted from Melilotus officinalis (L.) Pall and has been used as a traditional Chinese herb with heat-clearing and detoxifying functions (Hsia et al., 2019; Sun et al., 2020). Recent studies have explored the bioactivity of DIC, including its antibacterial, antiviral, anticoagulant, and antitumour properties. In medicine, it is used as a blood-thinning drug and as an anticoagulant rodenticide (Wallin et al., 2008; Rehman et al., 2013; Lata et al., 2015). However, DIC also has several disadvantages, such as poor water solubility and potential side effects, which limit its application (Keller et al., 1999; Díaz-Véliz et al., 2004; Li et al., 2015).

Porcine reproductive and respiratory syndrome (PRRS) is a prevalent infectious disease caused by porcine reproductive and respiratory syndrome virus (PRRSV) that results in considerable losses to the global swine industry. The infection manifests as reproductive failure in sows and respiratory distress in pigs of all ages. PRRSV is an enveloped, positive-sense single-stranded RNA virus that belongs to the family Arteriviridae in the order Nidovirales (Kappes and Faaberg, 2015). Currently, there is no effective or satisfactory treatment for PRRSV, and vaccination is the primary strategy for virus prevention. Despite the widespread use of various vaccines, they still have limitations in terms of safety and effectiveness, leading to occasional outbreaks of PRRS (Binjawadagi et al., 2014).

In this study, we synthesized a highly biocompatible and water-soluble form of GQDs using the active ingredient of a Chinese herbal medicine, DIC. This polymer combines the high biocompatibility of GQDs with the excellent antiviral properties of DIC. Our findings indicate that DIC-GQDs effectively inhibit PRRSV infection in both Marc-145 cells and porcine alveolar macrophages (PAMs) in a dose-dependent manner. Additionally, we demonstrated that the inhibition of PRRSV by DIC-GQDs is associated with the activation of the Janus kinase (JAK)/ signal transducer and activator of transcription (STAT) signaling pathway and the expression of IFN-β. These results provide valuable insights into the potential clinical application of DIC-GQDs as drug candidates for PRRSV infection treatment.

## 2 Materials and methods

### 2.1 Cell lines, viruses and reagents

Marc-145 cells, an African green monkey kidney epithelial cell line that is susceptible to PRRSV, were grown in Dulbecco’s modified Eagle medium (DMEM; Gibco) supplemented with 10% fetal bovine serum (FBS; Biological Industries) and 1% penicillin-streptomycin (Invitrogen) at 37°C in an incubator containing 5% CO2.

Porcine alveolar macrophages (PAMs) were extracted from the alveoli of 6-week-old not infected with PRRSV, PRV(Pseudorabies virus), or PCV2(Porcine circovirus 2) pigs. The cells were cultured in RPMI-1640 (Gibco) supplemented with 10% fetal bovine serum (FBS; Biological Industries) and 1% penicillin-streptomycin (Invitrogen) at 37°C in a humidified atmosphere containing 5% CO_2_.

The highly pathogenic PRRSV strain BB0907 (GenBank accession no. HQ315835.1) was used in all of the experiments and was designated PRRSV throughout this article. The NADC30-like PRRSV strain BJ2021 (GenBank accession no. OK095299.1), the classical PRRSV-2 strain CH-1a (GenBank accession no. AY032626.1), and the PRRSV-1 isolate SS-6 (GenBank accession no. GQ183803.1) were also used and are mentioned specifically by name.

DIC (Macklin, purity > 99%) was used for the *in vitro* experiments. Ribavirin (MCE), a clinically recognized broad-spectrum antiviral drug, was used as a positive control.

GQDs (XFNANO) were used for the *in vitro* experiments, and DIC-GQDs were synthesized for the *in vivo* experiments.

DIC, GQDs, DIC-GQDs, and ribavirin were dissolved in dimethyl sulfoxide (DMSO), and the final concentration of DMSO in the culture medium was less than 0.3%.

### 2.2 Synthesis and characterization of DIC-GQDs

DIC and GQDs were formed into DIC-GQDs by electrostatic adsorption by the XFNANO company. Briefly, the surface of GQDs was covered by DIC through electrostatic adsorption since GQDs and DIC have positive and negative charges, respectively. Ten milliliters of graphene oxide quantum dots (1 mg/mL) was mixed with 3 mL of aqueous bicoumarin solution (33 mg/mL, sodium hydroxide solution pH = 9) and stirred overnight, after which the reaction mixture was transferred to a regenerated cellulose dialysis bag (500–1000 Da) for dialysis to remove the free bicoumarin. After three days of dialysis, the internal dialysate was dried in a freeze dryer to obtain the dried GQD-bicoumarin powder, which was finally resuspended in 10 mL of ultrapure water (1 mg/mL). The DIC-GQD samples were characterized using various techniques, including transmission electron microscopy (TEM), UV-vis absorption, dynamic light scattering (DLS), Fourier transform infrared spectroscopy (FTIR) and thermogravimetric analysis (TGA).

### 2.3 Cell viability assay

The cytotoxicity of DIC was assessed using a CCK-8 assay. A total of 5 * 10^4^ Marc-145 cells per well were seeded in 96-well plates and incubated at 37°C for 36 h. The culture solution was then removed, and different concentrations of DIC solution diluted with DMEM were added and incubated at 37°C for 48 h. After discarding the culture medium, 10 μL of CCK-8 solution and 200 μL of cell culture medium were added to each well and incubated for an additional 2 h at 37°C. The 50% cytotoxic concentration (CC_50_) of DIC on Marc-145 cells was determined by measuring the OD450 values of each group using a microplate reader and GraphPad 9.0.

### 2.4 Anti-PRRSV activity assay

An antiviral activity assay was also conducted to compare the *in vitro* PRRSV-inhibiting capacities of the DICs and DIC-GQDs. Marc-145 or PAM cells were grown in 96-well or 24-well plates and infected with PRRSV at a multiplicity of infection (MOI) of 0.1 in essential media at 37°C for 1 h. Following the removal of the viral inoculums, fresh maintenance medium containing different concentrations of drugs was added. DMSO was used as the negative control, and ribavirin was used as the positive control. Cells and supernatants were collected at specified time points post infection. The virus titre was determined by an endpoint dilution assay and is expressed as log10 50% Tissue culture infective dose(TCID_50_)/1 ml. The viral nucleocapsid protein(NP) and Open Reading Frame(ORF)7 mRNA levels were determined by Western blotting and Quantitative real time polymerase chain reaction (qRT-PCR), respectively. Additionally, the cells were subjected to PRRSV-infected cell counting by immunofluorescence assay (IFA), and the Half maximal inhibitory concentration(IC_50_) was calculated by plotting the relative percentage of infected cells against the drug concentration.

### 2.5 Real-time RT-PCR

Marc-145 cells were collected by adding 200 μL of TRIzol to each well. Total RNA was then extracted from the cells using a Total RNA Extraction Kit, and the RNA was reverse transcribed using a HiScript II 1st Strand cDNA Synthesis Kit. The resulting cDNA was used as a template for qPCR following the instructions of the qPCR SYBR Green Master Mix. A negative control was included to obtain Ct values. The relative expression of the target genes was analyzed using the 2-ΔΔCt method (Maren et al., 2023). The assay was repeated three times for all treatment groups. The primer sequences for the genes can be found in Table 1.

**TABLE 1:** Primers used in this study.

Primer	Sequence (5′-3′)
PRRSV ORF7-F	AAACCAGTCCAGAGGCAAGG
PRRSV ORF7-R	TCAGTCGCAAGAGGGAAATG
β-actin-F	GTGATCTCCTTCTGCATCCTGTC
β-actin-R	CTCCATCATGAAGTGCGACGT
IFN-β-F	TGCTCTCCTGTTGTGCTTCTC
IFN-β-R	CTGCGGCTGCTTAATTTCCTC
ISG15-F	CACCGTGTTCATGAATCTGC
ISG15-R	CTTTATTTCCGGCCCTTGAT
ISG56-F	TCAGAGGTGAGA AGGCTGGT
ISG56-R	GCTTCCTGCAAGTGTCCTTC
IP10-F	TGCCATTCTGATTTGCTGCC
IP10-R	TGCAGGTACAGCGTACAGTT
MCP1-F	AGCCAGATGCAATCAATGCC
MCP1-R	GAACCCACTTCTGCTTGGGG
JAK1-F	ATGCATTTCTGCATCGAGCG
JAK1-R	ATTCAGCTGTCCAGCGTTCC
TYK2-F	TTCGGGGTGACCCTGTATGA
TYK2-R	GGACATTTGTCAGGTCGTGG
STAT1-F	TCTTCTGCCGGGTAGTTTCG
STAT1-R	CTCGAGGATGGCATACAGCA
STAT2-F	ACACCGTGGATGAGGCTTAC
STAT2-R	TAGCTTGGAAGGGACACACG

### 2.6 Western blot analysis

Total cellular proteins were extracted using RIPA lysis buffer containing protease inhibitors. Protein concentrations were determined using the BCA protein concentration assay kit from Beyotime. The protein concentration of each group was adjusted uniformly based on the BCA results. The protein samples were separated via electrophoresis via 12.5% polyacrylamide agarose gel electrophoresis (SDS-PAGE) and electroblotted onto nitrocellulose membranes. The target proteins were detected through blocking, incubation with primary antibody, and incubation with secondary antibody. The primary antibodies used were N-protein (1:1500), anti-β-actin (1:2000; Proteintech, USA), anti-JAK1 (1:1500; Abmart, China), anti-TYK2 (1:1500; Abmart), anti-STAT1 (1:1500; Abmart), and anti-STAT2 (1:1500; Abmart).

### 2.7 Indirect IFA

For immunostaining, PRRSV-infected or uninfected cells were fixed with 4% paraformaldehyde for 10 min. Subsequently, the cells were permeabilized with 0.25% Triton X-100 for 10 min at room temperature, followed by blocking with 1% bovine serum albumin (BSA) for 60 min at room temperature. The cells were then incubated overnight at 4°C with a mouse monoclonal antibody against the PRRSV NP. After three washes with PBS, the cells were incubated for 1 h at room temperature with a 1:1000 dilution of Alexa Fluor 488-coupled goat anti-mouse secondary antibody. The cell nuclei were restained with 3 ml of diamidino-phenyl-indole(DAPI). Immunofluorescence images were captured using a Leica DMI 4000B fluorescence microscope (Leica, Wetzlar, Germany). In each image, blue and green fluorescent spots were counted as the total number of PRRSV-infected cells and the number of PRRSV cells, respectively. The infection rate was calculated as the percentage of infected cells relative to the total cell count. The relative percentage of infected cells was determined by comparing the infection rate in the DIC-treated group to that in the DMSO-treated control group. The IC_50_ (the concentration required to protect 50% of cells from PRRSV infection) was determined by plotting the relative percentage of infected cells against the total concentration and calculated using GraphPad Prism 9.0 software.

### 2.8 Virus titration

Marc-145 cells were cultured in 96-well plates and infected with 10-fold serial dilutions of PRRSV. After the cells were incubated at 37°C for 1 h, the medium was replaced with fresh DMEM-2% FBS. Viral titres were determined 5 days after inoculation using endpoint dilution analysis. The 50% tissue culture infectious dose (TCID_50_) was calculated using the Reed–Muench method (Thakur and Fezio, 1981).

### 2.9 Virus adsorption assay

Marc-145 cells were cultured in 24-well plates at 4°C for 1 h. Subsequently, the cells were washed with precooled PBS. The culture supernatant was then replaced with a mixture of DIC-GQDs or DMSO and PRRSV (0.1 MOI) at 4°C, followed by incubation for another hour at 4°C. Afterwards, the cells were washed again with precooled PBS. Finally, the mRNA levels of PRRSV ORF7 in the cells were measured using qRT-PCR.

### 2.10 Virus internalization assay

Marc-145 cells were cultured in 24-well plates. Prior to treatment, the cells were pretreated with cycloheximide (CHX), a protein synthesis inhibitor, at a concentration of 10 μg/mL for 12 h. The cell surface was washed with precooled PBS, inoculated with PRRSV (1 MOI) and incubated at 4°C for 1 h. Afterwards, the cells were washed three times with precooled PBS to remove free virus particles. DMEM containing DIC-GQDs or DMSO was then added, and the mixture was incubated at 37°C for 30 min. To remove uninternalized virus, the cells were washed with citrate buffer (pH 3). Finally, the intracellular PRRSV ORF7 mRNA level was measured using qPCR.

### 2.11 Viral replication assay

Marc-145 cells were cultured on 24-well plates and inoculated with PRRSV at an MOI of 1. After incubating at 37°C for 6 h, the cells were washed three times with PBS. Then, fresh DMEM (with 2% FBS) containing DIC-GQDs or DMSO was added, and the mixture was incubated at 37°C. The intracellular PRRSV ORF7 mRNA level was measured via qPCR at 7, 8, 9, and 10 h.

### 2.12 Virus release assay

Marc-145 cells were cultured on 24-well plates. The cells were then inoculated with PRRSV at an MOI of 1, and the viral solution in the wells was aspirated. DMEM containing 2% FBS was added to the wells. At 18 h post infection (hpi), the cells were washed three times with PBS. Following the wash, DMEM containing DIC-GQDs or DMSO (both containing 2% FBS) was added, and the mixture was incubated at 37°C for 10, 30, or 60 min. After the indicated incubation times, the supernatants were harvested. The viral mRNA levels present in the cell supernatants was then determined using absolute fluorescence qPCR.

### 2.13 Direct PRRSV-DIC-GQD interaction

In order to explore the direct interaction between DIC GQDs and viruses, PRRSV (MOI of 0.1) was combined with various concentrations of DIC GQDs or ribavirin in essential media. The resulting mixture underwent an incubation period at 37°C for 1 h. Post-incubation, PRRSV and the medication were separated via ultrafiltration centrifugation. The mixture was introduced into a 0.5 ml ultrafiltration tool with a 30 kDa cutoff and then centrifuged at 7500 × g for 10 min at 4°C. The PRRSV particles trapped in the ultrafiltration membrane were rinsed twice with the 2%DMEM to eliminate any leftover compounds. Following this, the pellets were reconstituted in 2% DMEM and utilized to infect Marc-145 cells in 24-well plates for 1 h. After the cells were washed thrice with PBS, they underwent an additional 48-h incubation in fresh medium at 37°C. The count of PRRSV-infected cells was ascertained by analyzing viral mRNA levels through RT-PCR.

### 2.14 Time-of-addition experiment

DIC-GQDs were added to cells from 7 different delivery groups. The G1 group served as the negative control without DIC-GQDs. The G2 group was composed of cells treated with DIC-GQDs throughout the infection process of PRRSV. The G3 group was composed of cells exposed to PRRSV pretreated with DIC-GQDs and DIC-GQDs during virus adsorption. The G4 group was composed of cells treated with DIC-GQDs during virus adsorption. The G5 group was composed of cells treated with DIC-GQDs during virus invasion. The G6 group was composed of cells treated with DIC-GQDs during the viral replication stage. The G7 group was composed of cells that were pretreated with DIC-GQDs, followed by incubation in fresh medium for 24 h. qPCR was performed to measure the mRNA levels of PRRSV ORF7, while Western blotting was performed to detect the NP levels.

### 2.15 RNA isolation, sequencing and transcriptomic functional analysis

Total RNA was extracted from Marc-145 cells using TRIzol reagent after treatment with DIC-GQDs or DMSO for 24 h. The purity, concentration, and integrity of the RNA were assessed using a NanoPhotometer spectrophotometer, Qubit2.0 Fluorometer, and Agilent 2100 bioanalyzer. Eukaryotic mRNA was enriched with poly(A) tails using magnetic beads with oligo(dT) and then fragmented using ultrasound. The fragmented mRNA served as a template for synthesizing the first strand of cDNA using random oligonucleotides as primers in the M-MuLV reverse transcriptase system. The RNA strand was degraded with RNaseH, and the second strand of cDNA was synthesized with dNTPs using the DNA polymerase I system. The purified double-stranded cDNA was end-repaired, A-tailed, and connected to a sequencing junction. cDNA fragments of approximately 200 bp were selected using AMPure XP beads and PCR amplified, and the PCR products were purified again using AMPure XP beads. The final library was sequenced on an Illumina HiSeq™ 2000 sequencer. Gene expression levels were analyzed using DESeq2, with thresholds of absolute fold change (| log2-fold change|) > 1 and *p*-values < 0.05 to identify differentially expressed genes (DEGs). Finally, GO and KEGG analyses were performed to determine the effects of DIC-GQDs on cell biological processes, molecular functions, and cellular composition.

### 2.16 Animal experiments

Fifteen 5-week-old piglets, consisting of 8 boars and 7 sows, were selected for the study. These piglets were confirmed to be free of PRRSV, porcine circovirus type 2, swine fever virus, pseudorabies virus, swine influenza virus, and Mycoplasma hyopneumoniae. They were then randomly assigned to 3 different groups. Each group consisted of 5 piglets. The groups were as follows: i) the PRRSV infection and vehicle (corn oil) treatment group; ii) the PRRSV infection and DIC-GQDs (20 mg/kg) treatment group; and iii) the uninfected and untreated mock group. The piglets were challenged with the PRRSV strain BB 0907 via intranasal (1 mL) or intramuscular (1 mL, in the right neck) routes, with a viral titre of 3 × 10^5^ TCID_50_. After 24 h of infection, the piglets were orally administered 20 mg/kg body weight of corn oil containing DIC-GQDs or PBS. This dosing regimen was repeated every 3 days for a total of 14 days. The piglets’ general health and rectal temperature were monitored daily following infection. Serum samples and nasal swabs were collected at 1, 4, 7, 10, and 14 days post infection (dpi). On day 14, all the piglets were sacrificed, and serum, lung, and spleen tissues were collected for determination of the viral RNA load and histopathological analysis. This animal experiment was approved by the Animal Experiment Ethics Committee of Beijing University of Agriculture.

### 2.17 Ethics statement

All animal experiments were performed in accordance with the National Guidelines for Housing and Care of Laboratory Animals (China) and with the agreement of Beijing University of Agriculture’s Institutional Animal Care and Ethics Committee (approval No. SYXK2019-0005). All piglets were housed in Beijing University of Agriculture’s animal facility (Beijing, China).

### 2.18 Statistical analysis

All of the experiments in this study were independently repeated at least 3 times, and the data are expressed as the mean ± standard deviation (SD). Statistical significance was determined using Student’s t test for comparisons between two groups and one-way analysis of variance (ANOVA) for comparisons between more than two groups. *P* < 0.05 (*) indicates a significant difference, while *P* < 0.01 (**), *P* < 0.001 (***), and *P* < 0.0001 (****) indicates a highly significant difference.

## 3 Results

### 3.1 DIC inhibits PRRSV infection *in vitro*

**FIGURE 1:**
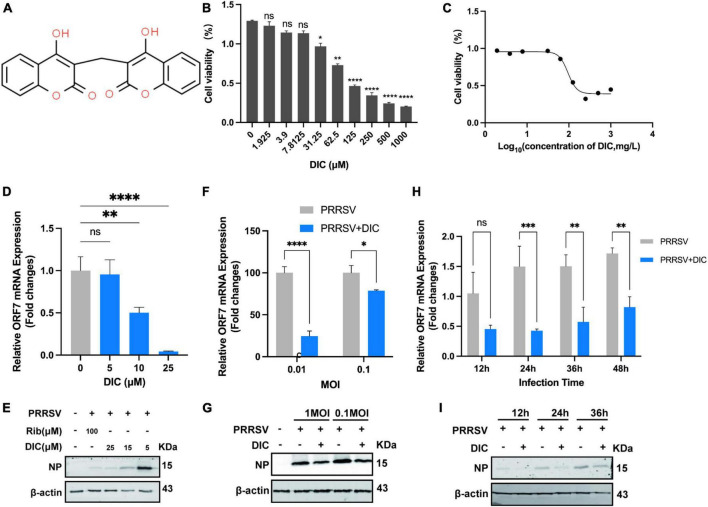
Cytotoxicity and anti-PRRSV activity of DIC in Marc-145 cells. **(A)** The chemical structure of DIC. **(B)** The cytotoxicity of DIC was assessed in Marc-145 cells using the CCK-8 assay after 48 h of incubation. The results are expressed as the cell viability of treated cells compared to that of control cells (set as 100%). **(C)** CC_50_ curves of DIC. Marc-145 cells grown in 24-well plates were infected with PRRSV (MOI of 0.1) for 1 h at 37°C and then cultured in fresh media containing various concentrations of DIC. At 48 h postinfection (hpi), the samples were subjected to RT-PCR **(D)** and Western blotting **(E)**. Marc-145 cells grown in 24-well plates were infected with PRRSV (MOI of 0.01 or 0.1) for 1 h at 37°C and then cultured in fresh media supplemented with DIC (25 μM). At 48 hpi, the samples were subjected to RT-PCR **(F)** and Western blotting **(G)**. Marc-145 cells grown in 24-well plates were infected with PRRSV (MOI of 0.1) for 1 h at 37°C and then cultured in fresh media supplemented with DIC (25 μM). At 12, 24, 36, and 48 hpi, the samples were subjected to RT-PCR **(H)** and Western blotting **(I)**. *P < 0.05; **P < 0.01; ***P < 0.001; ****P < 0.0001 compared to the respective virus control.

We conducted an experiment to assess the cytotoxicity of DIC in Marc-145 cells using a Cell Counting Kit-8 (CCK-8) assay (Figure 1A). Our results showed that DIC concentrations less than 31.25 μM did not significantly decrease the viability of Marc-145 cells (Figure 1B). However, when the concentration exceeded this threshold, DIC exhibited drug-dependent cytotoxicity in Marc-145 cells. The 50% cytotoxic concentration (CC_50_) of DIC on Marc-145 cells was 96.11 μM (Figure 1C). To further investigate the antiviral effect of DIC on PRRSV infection, we examined its impact on PRRSV ORF7 mRNA levels and NP levels in Marc-145 cells using qRT-PCR and Western blotting. We tested different concentrations of DIC (ranging from 5 to 25 μM) and used DMSO as a negative control. Our results showed that DIC, in a dose-dependent manner, significantly inhibited PRRSV ORF7 mRNA levels and NP levels at the same time points in Marc-145 cells, as indicated by the qRT-PCR (Figure 1D) and Western blot (Figure 1E) results, respectively. Considering the effect of different titres of PRRSV infection on the efficacy of DIC treatment, we treated Marc-145 cells infected with different titres of PRRSV (0.01 MOI and 0.1 MOI) using the same concentration of DIC. Our findings (Figures 1F, G) demonstrated that the PRRSV ORF7 mRNA levels and NP levels in the DIC-treated group were significantly lower than those in the DMSO group for both high and low titres of PRRSV. This finding suggested that DIC can effectively inhibit the proliferation of PRRSV. Next, we performed Western blotting and qPCR using the same concentration of DIC on Marc-145 cells infected with PRRSV at different time points (12, 24, 36, and 48 hpi). Our results (Figures 1H, I) showed that PRRSV ORF7 mRNA levels and NP levels increased from 12 hpi to 48 hpi. However, at the same hpi, compared with those in the DMSO group, the DIC-treated group exhibited significantly reduced PRRSV proliferation. This finding indicates that DIC treatment effectively reduces the proliferation of PRRSV.

### 3.2 Characterization of DIC-GQDs

**FIGURE 2:**
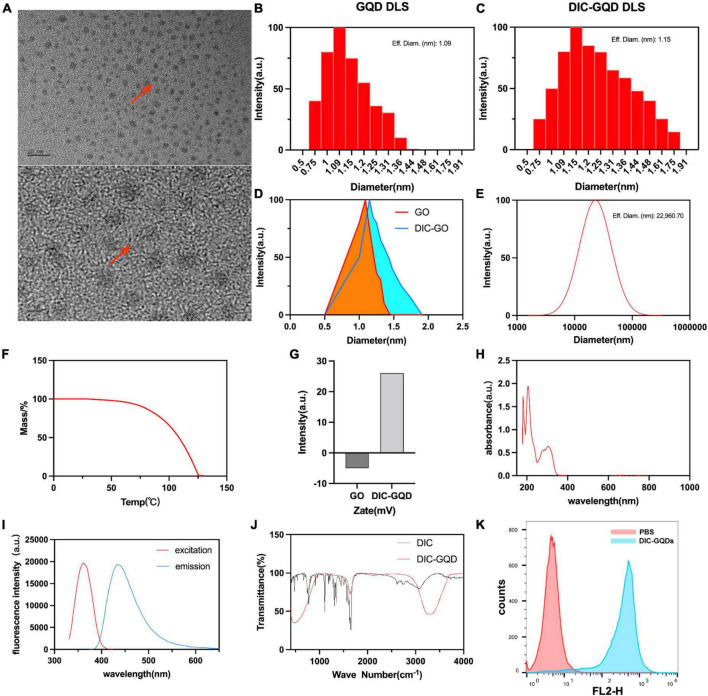
Characterization of DIC-GQDs. **(A)** TEM image of DIC-GQDs (scale bar = 2 nm and 10 nm). **(B–D)** DLS analysis of the GQDs and DIC-GQDs. **(E)** DLS analysis of DIC in aqueous solution. **(F)** TGA of DIC-GQDs. **(G)** Zeta potential analysis of DIC-GQDs. **(H)** UV–vis absorption of DIC-GQDs. **(I)** Fluorescence excitation (red) and emission (blue) spectra of the DIC-GQDs. **(J)** FTIR spectra of DIC and DIC-GQDs. **(K)** Marc-145 cells were treated with PBS and DIC-GQDs for 12 h. The cells were collected and analyzed via flow cytometry.

The size and surface morphology of the DIC-GQDs were characterized using high-resolution transmission electron microscopy (HR-TEM). The HR-TEM image in Figure 2A shows that the DIC-GQDs were round and well dispersed, with a uniform size distribution. The size distributions of the GQDs and DIC-GQDs, measured with a DLS analyser, are presented in Figures 2B, C. Consistent with the TEM image, the GQDs and DIC-GQDs exhibited high monodispersity, with average sizes of 1.09 and 1.15 nm, respectively. Notably, the particle size of the DIC-GQDs was slightly larger than that of the GQDs (Figure 2D). Additionally, we were interested in the particle size of DIC alone in aqueous solution. The average diameter of the DIC particles was 22960 nm (Figure 2E), which significantly hampers the water solubility of DIC.

Next, TGA was used to assess the stability of the DIC-GQDs. It was observed that the DIC-GQDs remained highly stable at normal body temperature but gradually decomposed at temperatures above 63.3°C (Figure 2F). The zeta potential, which is commonly employed to evaluate the stability of nanosystems, was measured. The zeta potential of the GQDs was found to be −5 ± 0.2 mV, and this value increased to 26 ± 0.31 mV in the presence of DIC-GQDs (Figure 2G). The UV-vis absorption and fluorescence spectra of the DIC-GQDs are presented in Figures 2H, I. The absorption peak at 260 nm was attributed to the π–π* transition, while the absorption peak at 350 nm was attributed to the n–π* transition. Notably, the DIC-GQDs exhibited a distinct blue fluorescence at 440 nm, with a maximum excitation wavelength of 365 nm. The functional groups of DIC-GQDs were characterized using FTIR, as shown in Figure 2J. The FTIR spectra revealed significant absorption peaks at 3278 and 1637 cm-1, corresponding to O-H and C - O, respectively, indicating the presence of -OH and -COO- groups on the surface of the DIC-GQDs. These results suggest that the DIC-GQDs retain certain functional groups. Moreover, flow cytometry analysis demonstrated that Marc-145 cells significantly absorbed DIC-GQDs (Figure 2K). The excellent stability and water solubility of DIC-GQDs under physiological conditions underscore their potential for medical applications.

### 3.2 DIC-GQDs inhibit PRRSV infection *in vitro*

**FIGURE 3:**
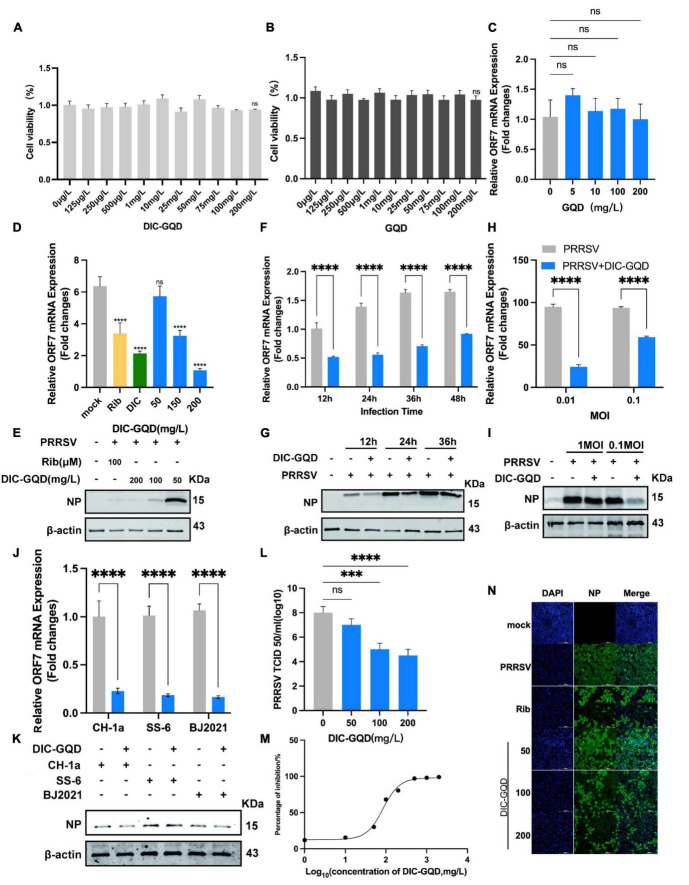
DIC-GQDs inhibit PRRSV replication in Marc-145 cells. **(A,B)** The cytotoxicity of DIC-GQDs and GQDs was evaluated in Marc-145 cells after 48 h of incubation using the CCK-8 assay. The results are expressed as the relative cell viability of treated cells compared to that of control cells (set as 100%). **(C)** Marc-145 cells were grown in 24-well plates, infected with PRRSV (MOI of 0.1) for 1 h at 37°C and subsequently cultured in fresh media supplemented with various concentrations of GQDs. At 48 hpi, the samples were subjected to RT-PCR. **(D,E)** Marc-145 cells grown in 24-well plates were infected with PRRSV (MOI of 0.1) for 1 h at 37°C and then cultured in fresh media supplemented with various DIC-GQD concentrations. At 48 hpi, the samples were subjected to RT-PCR and Western blotting. **(F,G)** Marc-145 cells grown in 24-well plates were infected with PRRSV (MOI of 0.1) for 1 h at 37°C and then cultured in fresh media supplemented with DIC-GQDs (200 mg/L). At 12, 24, 36 and 48 hpi, the samples were subjected to RT-PCR and Western blotting. **(H,I)** Marc-145 cells grown in 24-well plates were infected with PRRSV (MOI of 0.01 or 0.1) for 1 h at 37°C and then cultured in fresh media supplemented with DIC-GQDs (200 mg/L). At 48 hpi, the samples were subjected to RT-PCR and Western blotting. **(J,K)** Antiviral activity of DIC-GQDs against PRRSV infection by various strains (BJ2021, CH1a and SS-6) in Marc-145 cells was examined using RT-PCR and Western blotting at 48 hpi. **(L)** Cells grown in 6-well plates were infected with PRRSV (MOI of 0.1) for 1 h at 37°C and then cultured in fresh media containing various DIC-GQD concentrations. At 48 hpi, the samples were subjected to viral titre determination. **(M,N)** The antiviral activity of DIC-GQDs against PRRSV infection in Marc-145 cells was examined using IFA at 48 hpi. Cells were grown in 96-well plates, infected with PRRSV (MOI of 0.1) for 1 h at 37°C and subsequently cultured in fresh media supplemented with various DIC-GQD concentrations. IFA for the NP of PRRSV was performed at 48 hpi using an Alexa Fluor 488-conjugated goat anti-mouse secondary antibody (green). Nuclei were counterstained using 4,6-diamidino-2-phenylindole (DAPI) (blue). The results shown in panel C are the percentages of PRRSV-infected cells compared with those of the DMSO-treated control (absence of AS). Fig M shows the IC_50_ of DIC-GQDs, and Fig N shows the IFA image. Ribavirin (Rib) (140 mM) was used as the positive anti-PRRSV drug control. *P < 0.05; **P < 0.01; ***P < 0.001 compared to the respective virus control. Scale bar, 100 mm.

As with DIC, we evaluated the cytotoxicity of DIC-GQDs and GQDs in Marc-145 cells using a CCK-8 assay. Surprisingly, neither DIC-GQDs nor GQDs demonstrated significant cytotoxicity at a concentration of 200 mg/L (Figures 3A, B), indicating that their CC_50_ could not be determined. We initially investigated the antiviral effect of GQDs on PRRSV proliferation. However, the GQDs did not significantly affect PRRSV proliferation (Figure 3C), leading us to discontinue testing the effect of the GQDs on PRRSV. Subsequently, we assessed the antiviral effect of different concentrations of DIC-GQDs on PRRSV infection using qRT-PCR (Figure 3D) and Western blotting (Figure 3E). Like DIC, DIC-GQDs dose-dependently inhibited PRRSV proliferation *in vivo*, with a more pronounced effect than DIC. Furthermore, we treated Marc-145 cells infected with PRRSV with the same concentration of DIC-GQDs for different durations. Like DIC, DIC-GQDs significantly inhibited PRRSV proliferation at the same time points (Figures 3F, G). Our findings also revealed that DIC-GQDs downregulated the expression of PRRSV ORF7 mRNA and NP in Marc-145 cells infected with PRRSV strains of varying titres (Figures 3H, I). qRT-PCR and Western blot analysis demonstrated that DIC-GQDs effectively reduced the replication of the PRRSV BJ2021 strain, CH1a strain and SS-6 strain (Figures 3J, K). The TCID_50_ results further supported the dose-dependent inhibition of virus proliferation by DIC-GQDs (Figure 3L). Additionally, we assessed the anti-PRRSV activities of DIC-GQDs using IFA, and the results showed that DIC-GQDs exhibited similar anti-PRRSV activity (IC_50_, 84.82 mg/L) (Figures 3M, N). These findings indicate that, similar to DIC, DIC-GQDs significantly inhibit the proliferation of PRRSV.

### 3.3 DIC-GQDs suppress PRRSV infection via different treatment modes

**FIGURE 4:**
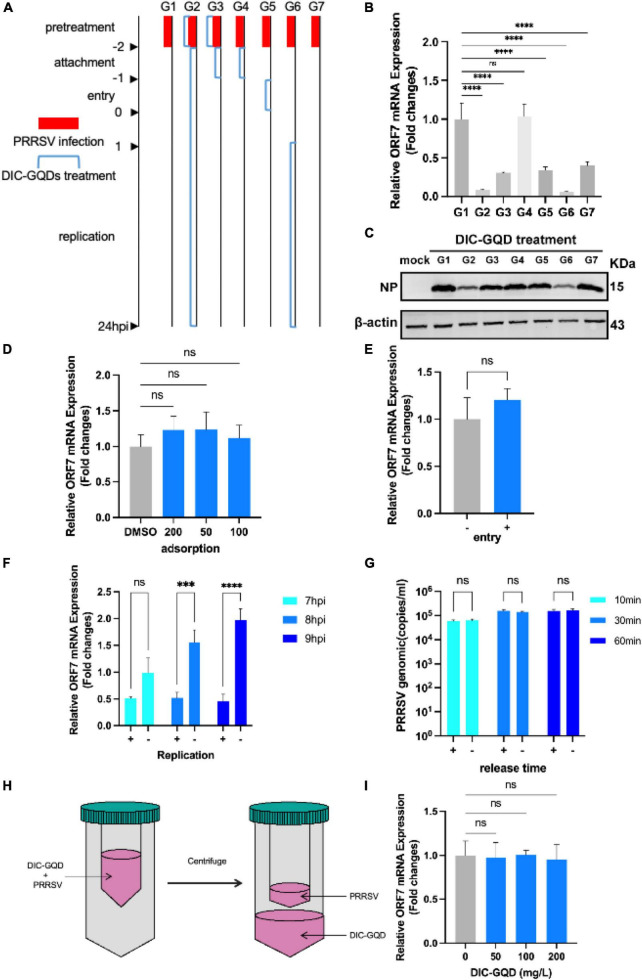
DIC-GQDs suppress virus proliferation by inhibiting the replication stage. **(A)** Marc-145 cells infected with PRRSV were treated with DIC-GQDs (200 mg/L) at different stages (G1-G7) of infection. The samples were subjected to RT-PCR **(B)** and Western blotting **(C)**. **(D)** Adsorption assay. Marc-145 cells were incubated with a mixture of DIC-GQDs or PBS and PRRSV for 1 h at 4°C, followed by harvesting for RT-PCR. **(E)** Internalization assay. Marc-145 cells were treated with CHX (10 μg/mL) for 12 h, incubated with PRRSV (0.1 MOI) for 1 h at 4°C, washed, and finally incubated with 200 mg/L DIC-GQDs or PBS for another hour at 37°C. The samples were subjected to RT-PCR. **(F)** Marc-145 cells were infected with PRRSV (0.1 MOI) for 6 h, after which the culture medium was replaced with 2% DMEM containing DIC-GQDs (200 mg/L) or PBS. At 7, 8, 9, and 10 hpi, the PRRSV levels were measured via RT-PCR. **(G)** Marc-145 cells were inoculated with 0.1 MOI PRRSV, and at 18 hpi, the cells were washed three times with DIC-GQDs or PBS. Afterwards, the cells were incubated for 10, 30 or 60 min, after which the cell supernatants were harvested. The amount of PRRSV present in the cell supernatants was quantified by absolute fluorescence. **(H)** PRRSV was incubated with DIC-GQDs at various concentrations in essential medium for 1 h at 37°C, after which the PRRSV was separated from the DIC-GQDs via ultrafiltration. **(I)** Recovered PRRSV was resuspended to infect Marc-145 cells. At 48 hpi, the cells and supernatants were harvested for the determination of viral mRNA levels via RT-PCR. ***P < 0.001; ****P < 0.0001.

To investigate the role of DIC-GQDs in different stages of PRRSV infection, we treated Marc-145 cells with DIC-GQDs after they were infected with PRRSV for different durations. The cells were divided into 7 groups: The G1 group represented the control group, the G2 group was the pretreatment phase + replication full cycle dosing group, the G3 group was the pretreatment phase + adsorption phase dosing group, the G4 group was the adsorption phase dosing group, The G5 group was the invasion phase dosing group, the G6 group was the replication phase dosing group, and the G7 group was the pretreatment phase dosing only group (Figure 4A). As shown in Figures 4B, C and the levels of NP and ORF7 mRNA in PRRSV were significantly lower in both the G2 and G6 administration groups than in the G1 control group. This finding suggested that DIC-GQDs may primarily exert antiviral effects in the later stages of the PRRSV replication cycle.

To further determine the impact of DIC on PRRSV infection, we investigated its role in the adsorption, internalization, replication, and release phases of the virus. Initially, Marc-145 cells were precooled for 1 h and then different concentrations of DIC-GQDs were added. The cells were then inoculated with PRRSV (1 MOI). A DMSO control group was also included. The cell plates were placed in a refrigerator at 4°C for 1 h. Subsequently, the cell surfaces were washed with prechilled PBS three times, and the intracellular PRRSV ORF7 mRNA level was measured via qRT-PCR. As shown in Figure 4D, DIC-GQDs had no effect on the adsorption of PRRSV. Next, the cells were pretreated with 10 μg/mL CHX for 12 h to inhibit intracellular protein expression and formation. The cells were then inoculated with PRRSV (1 MOI) and incubated at 4°C for 1 h. After the cells were washed with prechilled PBS to remove free virus particles, DIC-GQDs or DMSO was added, and the mixture was incubated for 30 min at 37°C. Subsequently, the cells were washed with citrate buffer (pH = 3) to remove uninternalized virus. The level of ORF7 mRNA in the PRRSVs that entered the cells was measured via qPCR. As shown in Figure 4E, DIC-GQDs had no effect on the entry phase of PRRSV. After Marc-145 cells were inoculated with PRRSV, the cells were collected at 7 hpi, 8 hpi, and 9 hpi to determine the relative mRNA expression of intracellular ORF7 (Figure 4F). The relative expression of ORF7 mRNA in the DIC-GQD-treated cells in the 8 hpi and 9 hpi groups was significantly reduced, indicating that DIC-GQDs inhibited the replication phase of PRRSV. To investigate the impact of DIC-GQDs on the release phase of PRRSV, Marc-145 cells were inoculated with 0.1 MOI PRRSV. At 18 hpi, the cells were washed three times with PBS, and DIC-GQDs were added. The mixture was incubated for 10, 30, or 60 min, after which the cell supernatants were harvested. The amount of PRRSV present in the cell supernatants was quantified using absolute fluorescence. The results showed no difference in viral levels between the DIC-GQD-treated group and the control group (Figure 4G), indicating that DIC-GQDs do not affect the release phase of PRRSV. Furthermore, we coincubated DIC-GQDs with the virus at 37°C for 1 h, resuspended the virus through ultrafiltration centrifugation and reinoculated the cells. After 36 h of cultivation, qRT-PCR was performed to measure ORF7 mRNA levels (Figure 4H). As shown in Figure 4I, DIC-GQDs were unable to directly kill the viruses. In conclusion, DIC-GQDs inhibited only the replication phase of PRRSV.

### 3.4 Transcriptional response of PAMs to DIC-GQD treatment

Given that PAMs are natural target cells in pigs, we aimed to determine the impact of DIC-GQDs on PRRSV-infected PAMs. To investigate the mechanism underlying the anti-PRRSV effect of DIC-GQDs, we conducted high-throughput RNA sequencing to analyze gene expression in PAM cells treated with 200 mg/L DIC-GQDs or DMSO for 24 h.

**FIGURE 5:**
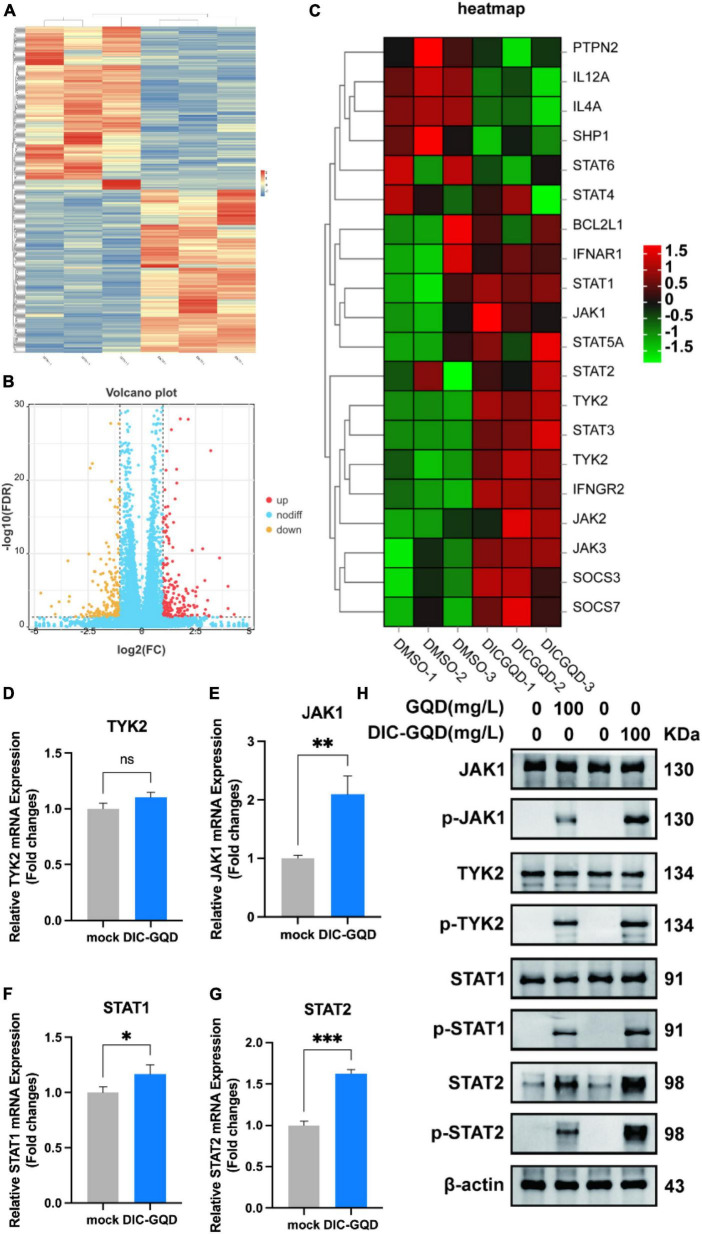
Transcriptional response of PAM cells to DIC-GQD treatment. **(A)** Unsupervised hierarchical cluster analysis was performed on the DEGs. The expression levels of DEGs are represented as FPKM-normalized log2-transformed counts. In the cluster analysis, low relative expression is indicated by the color blue, while high relative expression is indicated by the color red. **(B)** Volcano plot of upregulated and downregulated genes. **(C)** Heatmap depicting the expression of JAK/STAT-related genes in PAM cells subjected to modulation by either 200 mg/L DIC-GQDs or PBS treatment. In the heatmap, red and blue represent relative upregulation and downregulation, respectively. **(D–G)** qRT-PCR was performed to determine the expression of genes associated with the type I interferon signaling pathway following treatment with DIC-GQDs. **(H)** The protein levels of JAK1, p-JAK1, TYK2, p-TYK2, STAT1, p-STAT1, STAT2, and p-STAT2 were analyzed via Western blotting after treating PAM cells with DIC-GQDs and GQDs. *P < 0.05; **P < 0.01; ***P < 0.001.

According to the transcriptomic analysis, a total of 369 million clean reads were located within the Chlorocebus aethiops genome sequence, which accounted for 99.65% of the obtained clean reads. The Q30 base rate ranged from 93.30% to 94.77%, and each sample yielded between 45.6 million and 71.1 million clean reads. By using fragments per kilobase million (FPKM) mapped fragments, a total of 3,162,851 genes were found in the three groups. Figure 5A shows the number of differentially expressed genes (DEGs) based on the criteria of an absolute fold change (| log2-fold change|) greater than 1 and a p value less than 0.05. The genes were classified as upregulated if their transcripts were more abundant in cells treated with DIC-GQDs than in cells treated with DMSO. A total of 424 DEGs were identified, with 212 upregulated and 212 downregulated DEGs (Figure 5B). The top 100 DEGs indicated the activation of interferon-related signaling pathways. Consequently, we explored the networks associated with the type I interferon signaling pathway. Further analysis revealed that a considerable number of genes, including TYK2, JAK1, STAT1, STAT2, and IFNAR1, were associated with this pathway (Figure 5C). To validate the RNA-seq analysis results, qRT-PCR and Western blotting were performed to measure the expression of TYK2, JAK1, STAT1, and STAT2 in DIC-GQD-treated cells. The results (Figures 5D–H) revealed an increase in the expression levels of all four genes, consistent with the RNA-seq data. Collectively, these findings indicate that DIC-GQDs can activate the type I interferon signaling pathway.

### 3.5 DIC-GQDs inhibit PRRSV replication by activating the JAK/STAT signaling pathway

**FIGURE 6:**
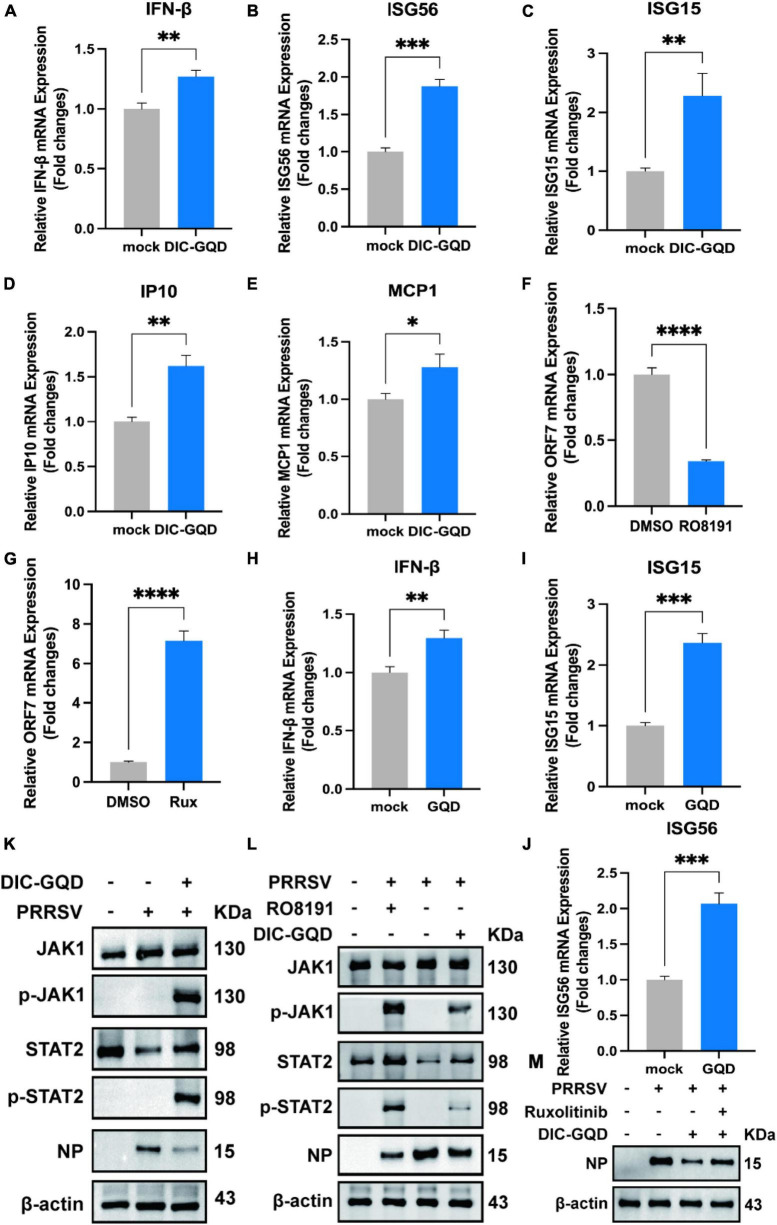
DIC-GQDs upregulate the JAK/STAT signaling pathway to inhibit PRRSV proliferation. **(A–E)** PAM cells were treated with 200 mg/L DIC-GQDs for 24 h. Subsequently, qRT-PCR was conducted to analyze the mRNA expression levels of IFN-β, ISG56, ISG15, IP10, and MCP1. **(F,G)** PAM cells were treated with RO8191 and ruxolitinib for 24 h. PRRSV ORF7 mRNA expression was detected via qRT-PCR. **(H–J)** PAM cells were treated with 200 mg/L GQDs for 24 h. Subsequently, qRT-PCR was conducted to analyze the mRNA expression levels of IFN-β, ISG15, and ISG56. **(K)** PAM cells were infected with PRRSV (MOI of 0.1) for 1 h. Afterwards, the cells were incubated in fresh medium containing DIC-GQDs for 24 hpi, after which the expression levels of JAK1, p-JAK1, STAT2, p-STAT2 and viral NPs were analyzed via Western blotting. **(L)** PAM cells were infected with PRRSV (MOI of 0.1) for 1 h. Afterwards, the cells were incubated in fresh medium containing DIC-GQDs and RO8191 for 24 hpi, after which the expression levels of JAK1, p-JAK1, STAT2, p-STAT2 and viral NP were analyzed via Western blotting. **(M)** PAM cells were infected with PRRSV (MOI of 0.1) for 1 h. Afterwards, the cells were incubated in fresh medium containing DIC-GQDs and ruxolitinib for 24 hpi, after which the expression level of viral NPs was analyzed via Western blotting. *P < 0.05; **P < 0.01; ***P < 0.001; ****P < 0.0001.

Type I interferons (IFNs) play a crucial role in activating immune response cascades and influencing the activation of both innate and adaptive immune responses. The typical signaling pathway for type I IFNs involves the activation of JAK and STAT, which in turn leads to the transcription of interferon-stimulated genes (ISGs) and the subsequent production of antiviral agents. According to our RNA-seq analysis, we observed significant upregulation of the JAK/STAT pathway. To investigate the impact of DIC-GQDs on the expression of type I interferons and their role in suppressing PRRSV, we treated PAM cells with 200 mg/L DIC-GQDs for 24 h and performed qRT-PCR for mRNA analysis. Our findings revealed that DIC-GQDs directly promoted the expression of IFN-β (Figure 6A). Additionally, as shown in Figures 6B–E, compared to the negative control, DIC-GQD treatment resulted in a noticeable increase in the mRNA expression levels of interferon-stimulated genes such as ISG15, ISG56, interferon inducible protein 10 (IP-10), and Monocyte chemoattractant protein-1 (MCP1). These ISGs are known to positively regulate the expression of IFN-β, thereby inhibiting viral infection and triggering various antiviral and proinflammatory reactions. Based on these results, DIC-GQDs may inhibit viral infection by modulating the mRNA expression levels of interferon-stimulated genes.

Using qRT-PCR, we investigated the anti-PRRSV activity of RO8191, a JAK/STAT pathway activator, and ruxolitinib, a JAK/STAT pathway inhibitor, in Marc-145 cells (Figures 6F, G). Our findings revealed that activation of the JAK/STAT pathway inhibits virus proliferation, while inhibiting this pathway promotes virus proliferation. To explore the impact of individual GQDs on the expression of type I interferon, we examined the expression of IFNβ, ISG15, and ISG56 (Figures 6H–J). Interestingly, we observed that treatment with GQDs alone could also enhance the expression of interferon. To validate the activation of the JAK/STAT pathway and the effect of DIC-GQDs on PRRSV proliferation, we conducted Western blotting. As depicted in Figure 6K, PRRSV-infected cells treated with DIC-GQDs exhibited a decrease in N-protein and a concomitant increase in p-JAK1 and p-STAT2, indicating activation of the JAK/STAT pathway and inhibition of the virus. Consistent with the qRT-PCR results, RO8191 activated the JAK/STAT pathway, as evidenced by the increased expression of p-JAK1 and p-STAT2. Additionally, RO8191 significantly inhibited PRRSV replication in PAM cells, and the effect of DIC-GQDs was similar to that of RO8191 (Figure 6L). Finally, we discovered that pretreatment with DIC-GQDs and ruxolitinib reversed the inhibitory effect of DIC-GQD treatment on PRRSV, suggesting that the JAK/STAT pathway is crucial for the anti-PRRSV activity of DIC-GQDs.

### 3.6 DIC-GQDs have therapeutic effects on PRRSV in piglets

**FIGURE 7:**
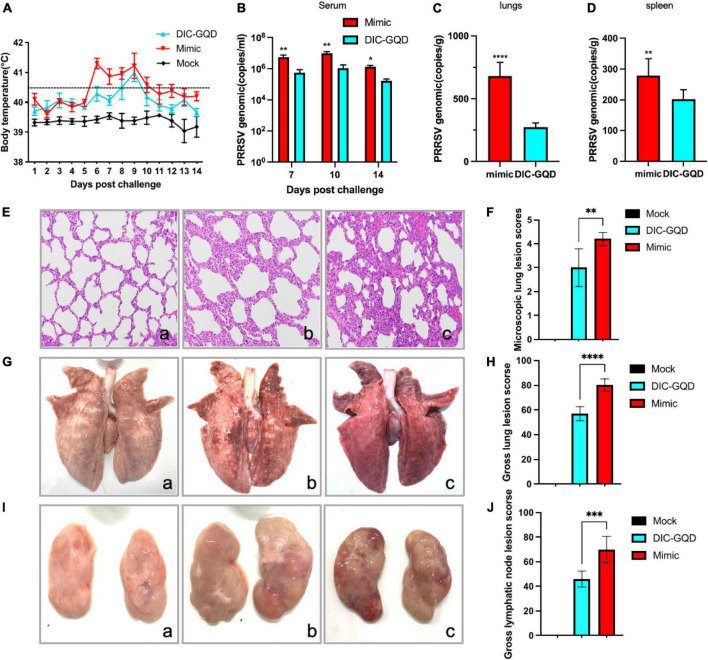
Therapeutic effect of DIC-GQDs in PRRSV-infected piglets. **(A)** Rectal temperatures of piglets from each group are presented as the mean ± SD (error bars). **(B)** The viral loads in the serum of pigs (log copies * ml-1), **(C)** lungs (log copies * ml-1) and **(D)** spleen were measured via qRT-PCR. **(E)** Microscopic observations of lungs collected from piglets. **(F)** Microscopic lung lesion scores. **(G)** Gross observations of lungs collected from piglets. **(H)** Gross lungs lesion scores. **(I)** Gross observations of lymphatic vessels collected from piglets. **(J)** Gross lymphatic lesion scores. *P < 0.05; **P < 0.01; ***P < 0.001; ****P < 0.0001.

Following challenge with virulent HP-PRRSV, all piglets in the vehicle control group exhibited a range of clinical signs, including high fever (≥ 40.5°C), appetence, lethargy, rough hair coat, dyspnoea, periocular oedema, and light diarrhea. Similarly, infected pigs treated with 200 mg/kg DIC-GQDs also experienced clinical fever during the experiment. However, their body temperature decreased by 0.3–1°C compared to that of the pigs in the vehicle control group (Figure 7A).

Blood samples were collected from the pigs at 7, 10, and 14 days postinfection (dpi). The copy number of the PRRSV genomic cDNA was determined using qRT-PCR analysis. In the serum of infected pigs treated with 200 mg/kg DIC-GQDs, the levels of circulating viruses were significantly lower than those in the vehicle control group (Figure 7B) (*p* < 0.05). Similarly, at 14 dpi, samples from the lungs and spleen of the infected pigs were collected. The copy numbers of PRRSV genomic DNA in the lungs and spleens of the infected pigs treated with 200 mg/kg DIC-GQDs were significantly lower than those in the vehicle control group (Figures 7C, D) (*p* < 0.05).

Necropsy examinations were also conducted to assess the lung lesions in the pigs. The vehicle group of pigs exhibited characteristics such as thickened alveolar septa, scattered hemorrhaging of alveolar septa, and increased numbers of inflammatory cells. Inflammatory exudation was also observed in the trachea of these pigs (Figure 7Eb). However, no pathological lesions were found in the control pigs (Figure 7Ea). The severity of microscopic lung lesions was significantly lower in the pigs treated with 200 mg/kg DIC-GQDs than in the vehicle control pigs (Figures 7Ec, F). Additionally, the lung tissues and lymphatic nodes of the infected pigs treated with vehicle showed significant lesions characterized by pulmonary consolidation. There were statistically significant differences in the gross lung and lymphatic node lesion scores between the vehicle group and the DIC-GQDs 200 mg/kg group (Figures 7G–J). The therapeutic effect of DIC-GQDs on piglets infected with PRRSV was observed in this study.

## 4 Discussion

In recent years, the emergence of nanomaterials has opened up new avenues for studying and applying biotechnology and medicine. These include bioimaging (Qu et al., 2022), biosensing (Fan et al., 2022), targeted drug delivery (Liang et al., 2014), and slow release (Kheirollahpour et al., 2020). Due to their water solubility, biocompatibility, low toxicity, and ease of modification, nanomaterials have been extensively researched for their potential to be combined with drugs. This combination aims to reduce side effects (Li et al., 2020), improve drug efficacy (Li et al., 2022), and enhance drug targeting (Huang et al., 2019). For instance, a study reported the synthesis of highly biocompatible glycyrrhizic acid nanoparticles (GANPs) using glycyrrhizic acid (GA) as a raw material. These nanoparticles were effective at inhibiting the proliferation of murine coronavirus (Zhao Z. et al., 2021). However, the inhibitory effect of GQDs on PRRSV has not been determined and requires further investigation.

Natural medicines and their active ingredients are gaining popularity among scientists worldwide (Liu H. et al., 2022). Studies have shown that these medicines have various beneficial effects, including antioxidative, anti-inflammatory, antiviral, antifungal, antitumour, and antimicrobial effects (Ben-Shabat et al., 2020). Dicoumarol (3,3′-methylenebis(4-hydroxycoumarin)), a coumarin-like compound, is an active ingredient found in Melilotus officinalis (L.) Pall (Sun et al., 2020). Initially, used as an anticoagulant, DIC was recently shown to have additional functions. It induces apoptosis in MCF-7 breast cancer cells without harming oocyte maturation or ovarian tissue in mice. DIC also acts as an inhibitor of NADPH quinone oxidoreductase 1 (NQO1) and has been found to inhibit HIV-1 replication by targeting NQO1 (Lata et al., 2015). These findings suggest that DIC has a wide range of biological activities. In our study, we synthesized DIC-GQDs by electrostatically adsorbing DIC onto graphene oxide quantum dots, addressing the issues of DIC’s water solubility and potential cytotoxicity. Our results demonstrated that both DIC and DIC-GQDs inhibited the proliferation of PRRSV in a dose-dependent and time-dependent manner. Interestingly, DIC-GQDs exhibit a greater antiviral effect than DIC alone. This can be attributed to the larger surface area and increased contact sites of DIC-GQDs, which enable enhanced interactions with viruses. Our findings are consistent with reports on glycyrrhetinic acid carbon quantum dots, which also demonstrate a greater antiviral effect on PRRSV proliferation than glycyrrhetinic acid (Tong et al., 2020). DIC-GQDs primarily target the replication stage of viral proliferation, but the specific antiviral mechanism has yet to be determined. To investigate the inhibitory mechanism of DIC-GQDs on PRRSV, we conducted a transcriptome analysis of PAM cells treated with DIC-GQDs. Our findings revealed the enrichment of several pathways associated with the JAK/STAT signaling pathway.

Interferon (IFN)-mediated innate immunity serves as the primary defense against pathogen invasion (Zhao J. et al., 2021). When pathogens are detected, IFN is produced and binds to cell surface receptors, initiating a signaling cascade through the JAK signaling sensor and activator of transcription (JAK-STAT). This cascade regulates more than 100 ISGs involved in transcription control (Lǚ et al., 2022). The JAK-STAT pathway plays a critical role in various biological processes, including cell proliferation, differentiation, apoptosis, immune regulation, and haematopoiesis (Bolli et al., 2003). Previous studies have shown that graphene oxide-silver nanoparticles can effectively upregulate the expression of IFN-α and ISGs, leading to the inhibition of PRRSV proliferation (Du et al., 2018). We discovered that DIC-GQDs can upregulate the expression of JAK1, STAT1, and STAT2 and enhance the phosphorylation of JAK1, TYK2, STAT1, and STAT2 in PAM cells in a dose-dependent manner, leading to the production of relevant ISGs. Moreover, the overexpression or induction of ISGs suppressed PRRSV replication in PAM cells. Our research revealed that DIC-GQDs can upregulate the expression of JAK1, STAT1, and STAT2 and promote the phosphorylation of JAK1, TYK2, STAT1, and STAT2 in PAM cells in a dose-dependent manner, leading to the production of relevant ISGs and the suppression of PRRSV replication in PAM cells.

As an active ingredient of traditional medicines, DIC plays an important role in anticoagulation, antibacterial, and antitumour effects. However, its poor water solubility, high cytotoxicity, and unclear mechanism of action limit its broad application. On the other hand, DIC-GQDs exhibit high biocompatibility and water solubility, which theoretically can mitigate the side effects of DIC. In conclusion, our study demonstrated that both DIC and DIC-GQDs effectively inhibited PRRSV replication and enhanced the body’s innate immune system. Furthermore, our findings demonstrate that DIC-GQDs exert their antiviral effects on PRRSV infection through the JAK/STAT signaling pathway, facilitating the development of novel antiviral strategies.

## 5 Conclusion

In this study, DIC-GQDs were synthesized through the electrostatic adsorption method using DIC, a natural drug, as the raw material. Our chemical characterization revealed that the DIC-GQDs were soluble in water and small in size. Moreover, the biological experiments demonstrated that DIC-GQDs possess effective antiviral activity against PRRSV. The antiviral mechanism of DIC-GQDs primarily involves the activation of the JAK/STAT signaling pathway, which enables PRRSV infection resistance. The animal experiments further confirmed that DIC-GQDs effectively alleviated clinical symptoms and pathological reactions in the lungs, spleen, and lymph nodes of PRRSV-infected pigs. Overall, this research provides novel insights into potential alternative treatments for PRRSV infection.

## Data availability statement

The original contributions presented in the study are publicly available. This data can be found here: NCBI (BioProject): https://www.ncbi.nlm.nih.gov/bioproject, accession number: PRJNA1116314.

## Ethics statement

The animal study was approved by the Beijing University of Agriculture’s Institutional Animal Care and Ethics Committee (approval No. SYXK2019-0005). The study was conducted in accordance with the local legislation and institutional requirements.

## Author contributions

ZL: Conceptualization, Data curation, Formal analysis, Investigation, Methodology, Project administration, Software, Supervision, Validation, Visualization, Writing – original draft. JJW: Writing – original draft. SW: Investigation, Writing – original draft. WZ: Investigation, Writing – original draft. XH: Writing – review and editing. JW: Writing – review and editing. HD: Writing – review and editing. SZ: Writing – review and editing. YG: Writing – review and editing. WY: Writing – review and editing. HL: Writing – review and editing. XL: Funding acquisition, Project administration, Resources, Writing – review and editing.
